# Contribution of Host Genetics to the Variation of Microbial Composition of Cecum Lumen and Feces in Pigs

**DOI:** 10.3389/fmicb.2018.02626

**Published:** 2018-10-31

**Authors:** Congying Chen, Xiaochang Huang, Shaoming Fang, Hui Yang, Maozhang He, Yuanzhang Zhao, Lusheng Huang

**Affiliations:** State Key Laboratory of Pig Genetic Improvement and Production Technology, Jiangxi Agricultural University, Nanchang, China

**Keywords:** gut microbiota, host genetics, heritability estimate, genome-wide association study, candidate gene, pigs

## Abstract

Pigs are a perfect model for studying the interaction between host genetics and gut microbiome due to the high similarity of gastrointestine and digestive system with humans, and the easily controlled feeding conditions. In this study, two pig populations which were raised in uniformed farm conditions and provided with the same commercial formula diet were used as the experimental animals. A systematical investigation of host genetic effect on the gut microbial composition was separately performed in porcine cecum lumen and feces samples through the comparison of microbial composition among full-sibs, half-sibs and unrelated members, heritability estimate (*h*^2^), and genome-wide association study (GWAS). The results showed that full-sib members had a higher similarity of microbial composition than unrelated individuals. A significant correlation was observed between the microbial composition-based kinship and the host SNP-based kinship in both populations (*P* < 9.9 × 10^-5^). We identified 81 and 67 microbial taxa having *h*^2^ > 0.15 in fecal and cecum luminal samples, respectively, including 31 taxa with *h*^2^ > 0.15 in both types of samples. GWAS identified 40 and 34 significant associations between host genomic loci and the abundance or presence/absence of bacterial taxa in the fecal and cecum luminal samples. Functional classifications of host candidate genes related to microbial taxa are mainly associated with metabolism, immunity functions and response, and signal transduction. The high similarity of heritable taxa and functional categories of candidate genes among pig, human and mouse suggests the similar mechanism of the host genetic effect on gut microbiome across mammalian species. The results from this study provided another evidence that host genetics contributes significantly to the gut microbiome.

## Introduction

Gut microbiota is a complex and heterogeneous ecosystem that has essential effects on host energy harvest (Turnbaugh et al., 2006), metabolism, immune development (Rooks and Garrett, 2016), and even behavior (Hsiao et al., 2013). Both environmental factors, such as diet, maternal seeding, lifestyle and medicine, and host factors, e.g., genetics, sex, aging, and disease state significantly influence the gut microbial composition (Costello et al., 2009; De Filippo et al., 2010; Goodrich et al., 2014, 2016b; Org et al., 2015a). Recent years, more and more studies have indicated the important role of host genetics in microbial community structure. The study in eight progenitor mouse strains of the collaborative cross observed that bacterial communities retained strain specificity although it became more similar after cohabitation of different strains of mice, suggesting an interaction of host genetics and environmental factors in shaping gut microbiota (Campbell et al., 2012). In humans, memberships with closer degree of relatedness in families showed a higher overall similarity in gut microbiome (Davenport, 2016). Monozygotic twin pairs had slightly more similar microbiomes compared to dizygotic twin pairs (Goodrich et al., 2014).

Heritability estimate facilitates to evaluate the strength of the host genetics on microbial composition. Goodrich et al. (2014) found that 5.3% of bacterial taxa had heritability estimate greater than 0.2 in stool samples of 416 pairs of dizygotic and monozygotic twins. The most heritable bacterial taxon was the family Christensenellaceae. This percentage was increased to 8.8% in their further study using 1,126 twin pairs (Goodrich et al., 2016a). Davenport et al. (2015) identified 15 heritable taxa in stools from different sampling seasons in Hutterites. Most of these heritable bacteria belong to the phyla Proteobacteria and Firmicutes. The study in mice of Hybrid Mouse Diversity Panel (HMDP) strains revealed that host genetic variation explains a substantial amount of the variation in gut microbial composition, which was up to 0.5 or more for many common taxa when experimental mice were maintained under controlled conditions (Org et al., 2015b). O’Connor et al. (2014) observed a wide range of heritability for taxa at the genus level (0.26–0.86) and identified the influence of genetic background in both microbial community structure and individual taxa in eight progenitor strains of the collaborative cross.

Cross-species studies have evidenced that gut microbiome is associated with host genetic variation (Campbell et al., 2012; Davenport, 2016; Goodrich et al., 2016b). Several studies were dedicated to identify host genes and variants contributing to the variation of microbial taxa by genome-wide association study (GWAS) or quantitative trait locus (QTL) mapping using quantitative measures of the microbiome as complex traits (O’Connor et al., 2014; Blekhman et al., 2015; Davenport et al., 2015; Org et al., 2015b; Bonder et al., 2016; Goodrich et al., 2016a; Turpin et al., 2016). For instances, Wang et al. (2016) identified multiple genetic loci, including *vitamin D receptor* associated with overall microbial variation and individual taxa. Goodrich et al. (2016a) discovered the associations between heritable taxa and genes related to diet, metabolism and olfaction, and found that the variants in or near the *ALDH1L1* and *LCT* were associated with the abundance of SHA-98 and *Bifidobacterium*, respectively. Eighteen QTLs were identified to link with relative abundances of microbial taxa in mice (Benson et al., 2010). However, all above studies relied on stool samples. Fecal microbiota is mostly from the colon and luminal microbes (Eckburg et al., 2005). The microbiota further up the gastrointestinal tract may be rarely detectable in the stool. Whether host genetics plays the similar effect on the microbiota of other intestinal tracts is unknown at present.

Pigs are a perfect animal model for studying the interaction between host genetics and gut microbiota due to the high similarity of gastrointestine and digestive system with humans. Compared to humans, pigs are always raised in uniform farm conditions that are easy to man-made control. All experimental pigs can be fed with the same commercial formula diets. Further, gut luminal samples from different intestine sites can be harvested at slaughter. However, to our knowledge, there is no study about host genetic effect on gut microbiome in pigs, especially with the samples from different gut compartments.

**FIGURE 1:**
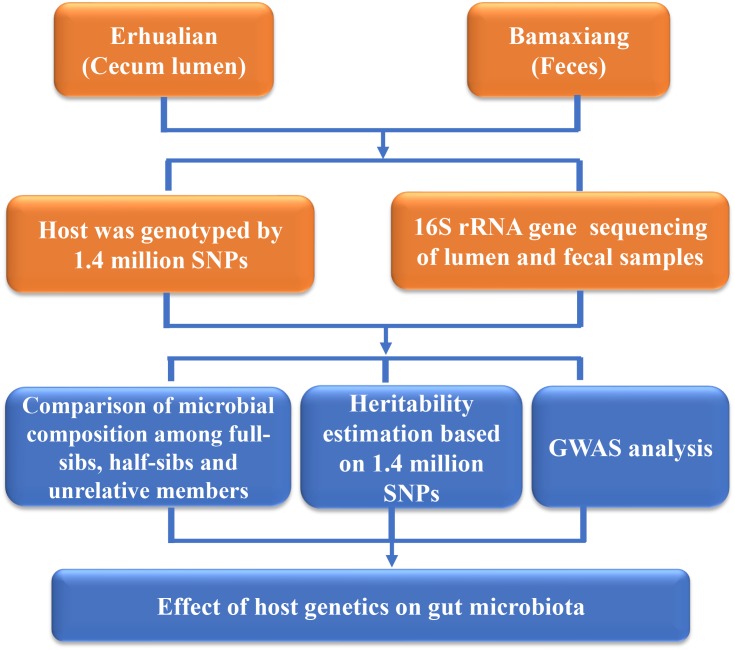
Flowchart depicting the experimental design for studying the host genetic effect on gut microbial composition in pigs.

In the current study, in order to evaluate the effect of host genetics on microbial composition of porcine cecum and feces, we determined the phylogenetic composition of microbial communities of porcine cecum lumen and feces using 16S rRNA gene sequencing. The contribution of host genetics toward the variation of microbial compositions of cecum and feces was evaluated through comparing the microbial diversity among full-sibs, half-sibs and unrelated members, estimating the heritability of the relative abundance of microbial taxa, and performing GWAS to identify the host genomic loci influencing the microbial composition (Figure 1).

## Materials and Methods

### Animals and Sample Collection

To evaluate the contribution of host genetics toward the variation of microbial community structure of different gut locations, a total of 500 samples were collected and used in this study, including 256 cecum luminal samples from Erhualian pigs (151 gilts and 105 castrated boars) and 244 fecal samples from Bamaxiang pigs (121 gilts and 123 castrated boars). Full siblings were co-housed with their mothers prior to weaning. After weaning, both populations were raised in the same farm house, where 7–8 pigs were randomly grouped into a 10 m^2^ open. All experimental pigs were fed two times a day by providing with the same corn-soybean based commercial formula diet, which was comprised of 16% crude protein, 3100 kJ digestive energy and 0.78% lysine. Water was available *ad libitum* from nipple drinkers. All boars were castrated at the age of day 60. All pigs were healthy and did not receive any antibiotic treatment within 2 months before slaughter. The experimental pigs were slaughtered at 300 ± 3 days after fasting but water-free overnight as described previously (Yang et al., 2016). The luminal contents of cecum were collected within 30 min after slaughter, and fecal samples were harvested from rectum before slaughter. All samples were immediately dipped in liquid nitrogen, and then transferred into -80°C freezer until use. All procedures involving animals were conducted according to the guidelines for the care and use of experimental animals established by the Ministry of Agriculture of China. Animal Care and Use Committee (IACUC) in Jiangxi Agricultural University also approved this study.

### DNA Extraction

The microbial DNA was extracted using the QIAamp Fast DNA stool mini kit (Qiagen, Germany) according to manual instructions. Host genomic DNA used for genotyping of high-density SNP chips was extracted from ear tissues of the tested pigs with the standard phenol–chloroform method. The quality and concentration of DNA samples were determined by Nanodrop-1000 and 0.8% agarose gel electrophoresis.

### 16S rRNA Gene Sequencing and Data Processing

The conserved primers 515F (GTGCCAGCMGCCGCGGTAA) and 806R (GGACTACHVGGGTWTCTAAT) were designed for amplifying the hypervariable V4 region of 16S rRNA gene under the annealing temperature of 56°C with 30 cycles. The products of amplification were purified, and then sequenced on an Illumina MiSeq platform (Illumina, United States). All 16S rRNA gene sequencing data were submitted to the NCBI database with accession number PRJNA348164. The sequence reads with ambiguous base, 10 consecutive same bases and low quality were filtered out. The chimeric sequences were further identified and discarded using USEARCH software (v7.0.1090) (Wang et al., 2007). The paired-end reads were assembled into tags based on the overlapped sequences with FLASH (v.1.2.11) (Magoc and Salzberg, 2011). Tags with >97% sequence similarity were clustered into operational taxonomic units (OTUs) using USEARCH software (v7.0.1090) (Wang et al., 2007). To obtain the taxonomic information, the 16S rRNA gene sequences were further assigned to the GreenGene database (Desantis et al., 2006) using RDP Classifier with an assignment cutoff of 0.8 (Cole et al., 2005). The microbial abundances at each taxonomic level were summarized using the QIIME (v 1.9) (Navas-Molina et al., 2013).

### Genotyping of Experimental Pigs

The experimental pigs were genotyped using the customized 1,400 k SNP Chips, which contained 1.4 million SNPs from both Chinese indigenous and Western commercial pig breeds according to the standard genotyping procedure (Affymetrix, United States). We performed quality control for the genotyping data with the thresholds of call rate ≥95%, minor allele frequency (MAF) ≥5% and Hardy Weinberg equilibrium (HWE) *P*-value > 5 × 10^-6^ using PLINK (v1.07) (Purcell et al., 2007). A final set of 751,802 and 731,411 SNPs were passed the quality control and used for further analyses in the Bamaxiang and Erhualian population, respectively. A total of 462 experimental pigs (227 Erhualian and 235 Bamaxiang) had both genotyping and 16S rRNA gene sequencing data.

### Statistical Analysis

#### Comparison of Microbial Diversity Among Full-Sibs, Half-Sibs, and Unrelated Pigs

Full-sibs from 40 pairs of parents and half-sibs from 53 boars in the Erhualian population were used to compare the microbial diversity of cecum lumen among full-sibs, half-sibs and unrelated individuals. The same analysis was also performed in the Bamaxiang population with full-sibs from 36 pairs of parents and half-sibs from 40 boars. To avoid the effect of cohabitation on the microbial diversity, the pigs used for the comparison of microbial diversity in each of full-sib, half-sib and unrelated pig groups were drawn from different pens. Weighted and Unweighted Unifrac distance between samples were calculated using the QIIME (v 1.9) (Navas-Molina et al., 2013), and compared by the Student’s *t*-tests with 1,000 Monte Carlo simulations.

Association of host genetics with gut microbial composition was further evaluated by calculating the correlation between host genetic kinship and microbial composition-based kinship among individuals. The formula: $Kinship=\frac{1}{p}\Sigma_{i=1}^{p}(x_{i}-1_{n}{\overset{¯}{x}}_{l}){\left(x_{i}-1_{n}{\overset{¯}{x}}_{l}\right)}^{T}$ was used for calculating the kinship matrix based on the host genotyping data and the microbial composition data (OTU compositions and relative abundances), respectively (Zhou and Stephens, 2012); where *x* represents the *n* × *p* matrix of genotypes or OTUs, *x_i_* and ${\overset{¯}{x}}_{l}$ represent the genotypes of *i*th and *l*th SNPs or relative abundances of *i*th and *l*th OTUs, and 1*_n_* means a *n* × 1 vector of 1’s. Mantel test was then used to evaluate the correlation between the host SNP-based kinship and microbial composition-based kinship.

#### Heritability Estimation

Heritability was estimated using the Bayesian sparse linear mixed model in the GEMMA software (v 0.94) (Zhou et al., 2013), following the formula *y* = 1*_n_*μ + Xβ + *u* + *e*. Where y is an n-vector of relative abundances of bacteria, μ is a scalar representing the mean of relative abundances of microbes, X is an *n* × *p* matrix of genotypes on *n* individual at *p* genetic marker, β is the corresponding *p*-vector of the genetic marker effect, and *e* is the residual error. In this method, the relative abundance of microbes was corrected for the effects of sex and sampling batch, and decomposed into SNP sparse effect Xβ, random effect *u* as well as residual error effect *e*. To estimate the chip heritability, 10^5^ times MCMC sampling method was used for the estimation of β, *u* and other hyper-parameters. The proportion of variance of microbial abundance (*h*^2^) explained by SNPs was calculated as follows: $PVE(\beta ,u,\tau )=\frac{\mathrm{var}\left(X\beta +u\right)}{\mathrm{var}(X\beta +u)+\tau^{-1}}$, where the τ^-1^ is the variance of the residual error. The lowest 2.5% and the highest 2.5% of the estimated values were removed to generate a 95% confidence interval of *h*^2^ for a given microbe.

#### GWAS Analysis

We performed GWAS analysis for relative abundance or presence/absence of OTUs and taxa using the method reported previously (Turpin et al., 2016) with the GenABEL package in R software (Aulchenko et al., 2007). Because many bacterial taxa and OTUs were not presented in many samples, we defined the taxa and OTUs that were presented in >95% samples as core OTUs and taxa, and those presented in 20∼95% of experimental pigs as random OTUs and taxa, while taxa or OTUs presented in less than 20% of samples were discarded from further analyses. We controlled for the effects of sex, sampling batch and the first three genetic principal components across all model. For core OTUs and taxa, a log-normal model on the log of the relative abundance for non-zero counts were used to estimate the associations of host genotypes with bacterial taxa by the qtscore function in the GenABEL package (Aulchenko et al., 2007). For random OTUs and taxa, we adopted a two-part log-normal model that accounted for both binary feature (detected/undetected) and quantitative feature (relative abundance) of microbes to perform the GWAS analysis as described by Turpin et al. (2016). In brief, the first part of the model used a logistic regression model on the absence/presence to detect the association between host SNPs and the binary feature of a microbe. In the second part, the non-zero relative abundance of each OTU or taxon was used for GWAS analysis in a log-normal model as described above. Genomic control (GC) was used to correct the effect of stratification (λ) that was estimated from the null test statistics (under the null hypothesis that no SNP was associated with the trait) (Devlin et al., 2001). We evaluated population stratification by examining the distribution of test statistics in a quantile-quantile (Q-Q) plot (Pearson and Manolio, 2008). We filtered out those results with λ < 0.99 or λ > 1.06. Bonferroni correction was used to adjust the multiple tests (Benjamini and Hochberg, 1995). The genome-wide significance threshold was set at 0.05/SNP numbers, and the suggestive significance level was determined by 1/SNP numbers. Linkage disequilibrium (*r*^2^) between SNP markers was estimated by PLINK (v1.07) (Purcell et al., 2007). The empirical confidence interval was determined by *r^2^* ≥ 0.8 between the strongest SNP and its surrounding SNPs.

#### Candidate Gene Annotation

To find candidate genes for all detected genomic loci above the suggestive significance level, the gene closest to the top SNP of each locus was searched based on the porcine reference genome assembly (Sscrofa10.2). Function capacities of the selected genes were then annotated using the online panther classification system (Mi et al., 2013), GeneCards and the Mouse Genome Database (MGI) (Eppig et al., 2015).

## Results

### Variation of Microbial Composition in Porcine Cecum Lumen and Feces

We determined the phylogenetic composition and the variability of microbial communities in a total of 500 samples from two Chinese pig populations, including 256 cecum luminal samples from Erhualian pigs and 244 fecal samples from Bamaxiang pigs using 16S rRNA gene sequencing. After quality control, for cecum luminal samples, an average of 36,324 (ranging from 19,356 to 62,376) clean reads per sample was obtained. The clean reads were assembled into 18,162 tags (Supplementary Table S1). Tag sequences sharing ≥97% pairwise similarity were clustered into OTUs. On average, we identified 718 non-singleton bacterial OTUs for each sample. OTUs were further assigned to obtain the taxonomic information, and 92 bacterial genera belonging to 13 phyla were detected (Supplementary Table S1). For fecal samples, we obtained an average of 61,669 (26,432∼102,350) clean reads per sample, which were assembled into 22,174 tags (Supplementary Table S1). The average numbers of OTUs, bacterial phyla and genera identified in fecal samples were 888, 15, and 117, respectively.

**FIGURE 2:**
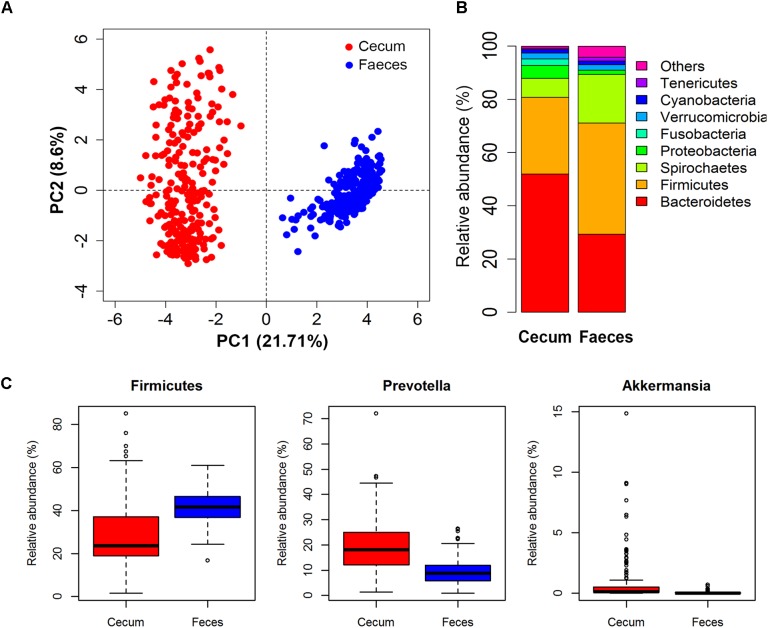
Comparison of the microbial composition between two types of samples and among individuals within population with 16S rRNA gene sequencing data. **(A)** PCA analysis showed great difference of microbial composition between cecum lumen and feces; **(B)** Comparison of microbial composition at the phylum level; and **(C)** Distribution of relative abundances of bacterial genera among individuals. Samples/bacterial genera are represented along the horizontal axis, and relative abundance is denoted by the vertical axis.

Microbial composition varied greatly between two types of samples (Figure 2A). Of the 92 and 117 bacterial genera identified in cecum lumens and stools, 73 genera were shared in both types of samples. In cecum lumen samples, Bacteroidetes was the most abundant bacterial phylum (51.92% ± 15.44%) and Firmicutes was ranked the second (28.81% ± 14.17%), while the relative abundance of Bacteroidetes and Firmicutes in feces was 29.32% ± 6.12% and 41.79% ± 7.38%, respectively (Figure 2B). The microbial composition was also distinct across animals. The relative abundances of Firmicutes across all pigs in the Erhualian ranged between 15.45 and 33.50%. In Bamaxiang pigs, it ranged from 16.75 to 63.19%. At the finer taxonomic level, even larger variation of bacterial structures was observed among pigs within population. For instances, *Prevotella* varied in the relative abundance from 0.61 to 72.07% across individuals of Erhualian pigs, and from 0.94 to 26.38% across Bamaxiang pigs. The relative abundance of *Akkermansia* ranged from 0 to 15.0% across Erhualian pigs, but only from 0 to 0.67% across Bamaxiang pigs (Figure 2C).

### Comparison of Microbial Diversity Among Full-Sibs, Half-Sibs, and Unrelated Pigs

**FIGURE 3:**
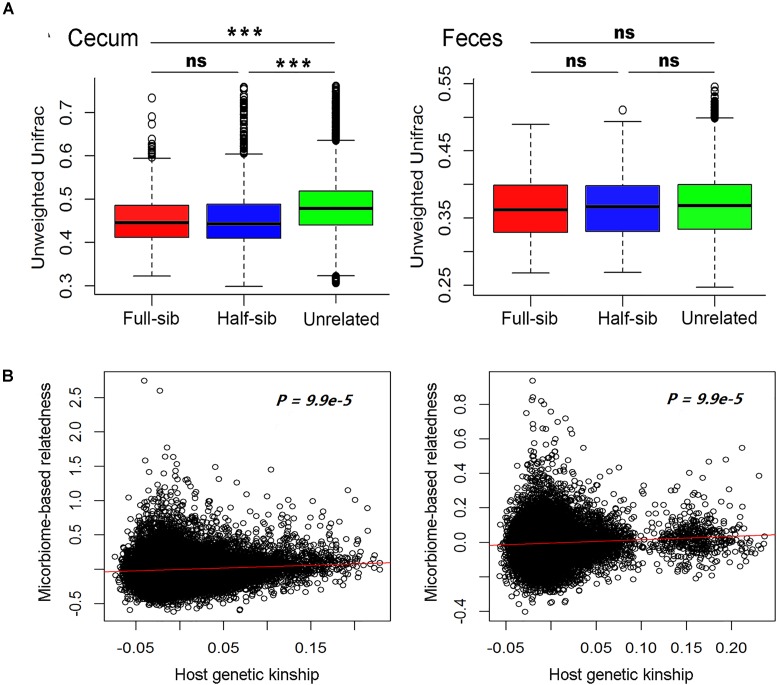
Host genetic effect on microbial compositions of cecum lumen and feces. **(A)** Comparison of the phylogenetic diversity of microbial composition among full-sib pairs, half-sib pairs and unrelated individuals by Unweighted Unifrac analysis. Full-sib pairs showed a higher similarity of microbial composition than unrelated individuals in both Erhualian (EHL) and Bamaxiang (BMX). The analysis was performed by QIIME (v 1.9). **(B)** Host genetic kinship calculated from host genome data (*x*-axis) is correlated with the microbial composition-based kinship (y-axis). In the panels, solid red lines represent a linear regression fit to the data.

We compared the diversity of microbial communities among full-sibs, half-sibs from the same boar and unrelated pigs in each of the two sample types. To avoid the effect of cohabitation on the diversity of gut microbiome, the pigs used for comparison in each of full-sib, half-sib and unrelated individual groups were drawn from different pens (see section Materials and Methods). As shown in Figure 3A and Supplementary Figure S1, full-sibs showed a higher similarity of microbial communities than unrelated pigs in both Weighted and Unweighted Unifrac distance analysis although the difference was not achieved significance level in feces samples. Full-sibs shared a pair of common parents, and to some extent, the shared microbiota might be partly resulted from the transfer of mother to offspring during the birth and nursing. However, the higher similarity of microbial composition was also observed within half-sibs than unrelated individuals. Half-sibs were born and nursed by different sows, so the maternal seeding effect was avoided. Furthermore, we calculated the kinships among experimental pigs based on the microbial compositions. Interestingly, a significant correlation was observed between the microbial composition-based kinship and the host genetic (SNP-based) kinship in both populations (*P* < 9.9 × 10^-5^, Figure 3B). These results suggest the host genetic effect on the diversity of gut microbiota.

### Heritability (*h*^2^) Estimation for the Relative Abundance of Bacterial Taxa

**FIGURE 4:**
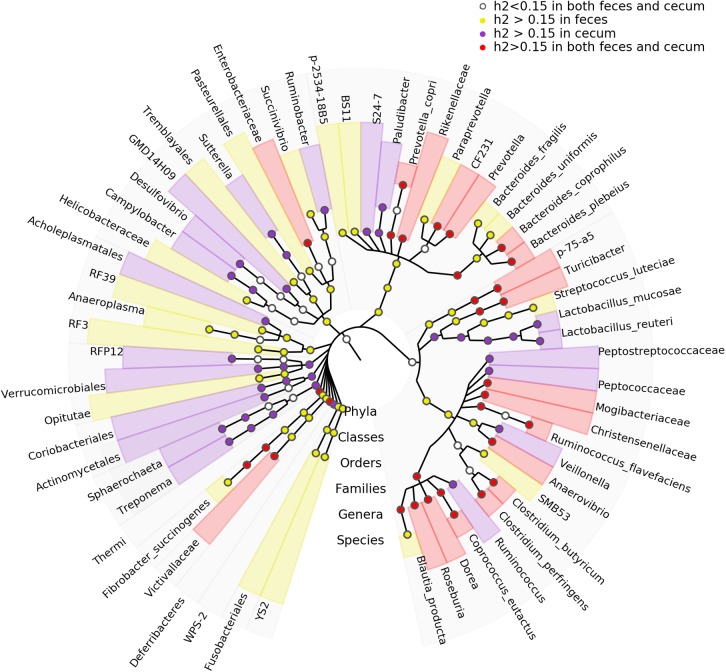
Taxonomic representation of heritable bacteria taxa. The central dot in cladogram represents kingdom; each successive circle moving outward is one step lower phylogenetically. The phylogenic relationships of the taxa were obtained by the RDP database (Release 11.4). The circles showing different colors represent the taxa with different strength of heritability in cecum lumen and feces as measured by *h*^2^.

We estimated the heritability of the relative abundance of bacterial taxa by SNP-based approach. We identified 81 and 67 microbial taxa showing *h*^2^ values > 0.15 in fecal and cecum luminal samples, respectively. The species *Blautia producta* (*h*^2^ = 0.41) showed the highest heritability in fecal samples, while the family Ruminococcaceae and the genus *Lachnospira* had the highest *h*^2^ values (*h*^2^ = 0.56) in cecum luminal samples (Supplementary Table S2). Furthermore, we identified 31 microbial taxa that had the *h*^2^ > 0.15 in both types of samples (Figure 4). Interestingly, *Blautia* has been reported to have the highest heritability value in pig feces samples by Camarinha-Silva et al. (2017).

**FIGURE 5:**
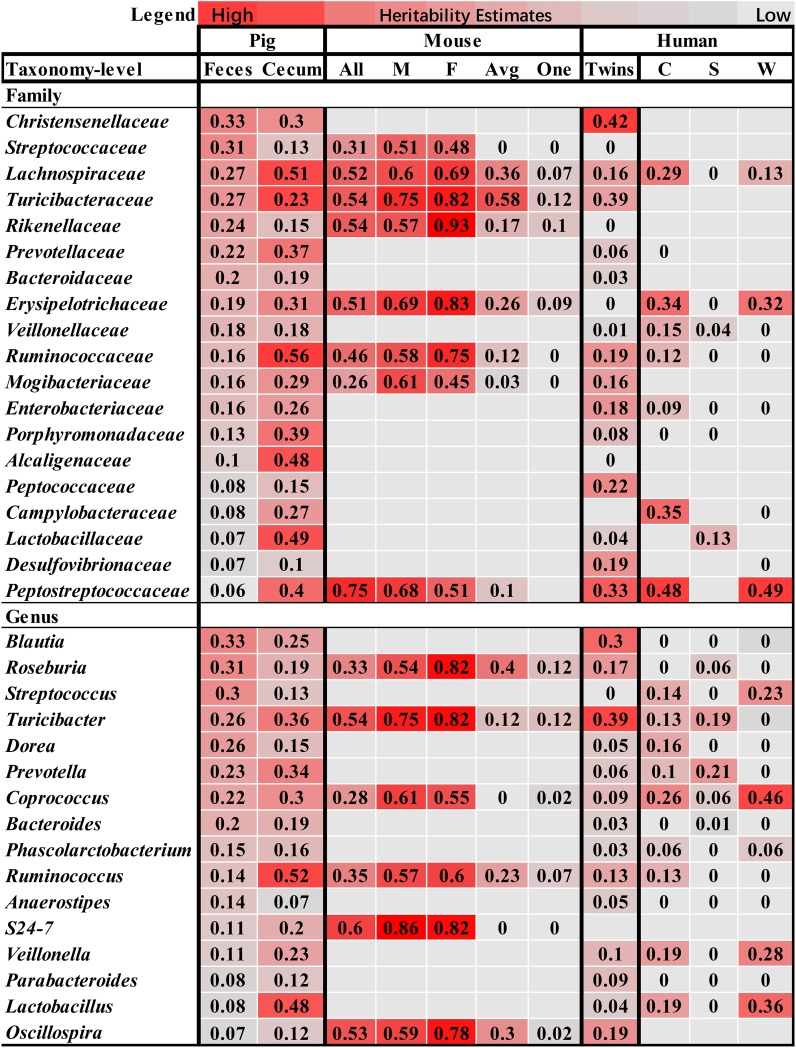
Comparison of the heritability of microbial taxa among pig, human and mouse. The color gradient over the heritability estimates ranges from the lowest heritability estimate (white) to the highest heritability estimate (red) in the given study. The heritability estimates in mouse were obtained from the studies reported by Org et al. (2015b), in which heritability was estimated using all mice (All), male (M), female (F), an average per strain (Avg) and a single mouse per strain (One), and O’Connor et al. (2014). The estimates in human were referred to the studies by Goodrich et al. (2016a), and Davenport et al. (2015), in which the heritability was estimated in the Winter (W), Summer (S), and seasons combined dataset (C).

Compared to the heritability estimation in humans and mice, we identified six bacterial families and three genera showing high *h*^2^ values in all of pigs, humans and mice, including Lachnospiraceae, Erysipelotrichaceae, Turicibacteraceae, Ruminococcaceae, Mogibacteriaceae, Peptostreptococcaceae, *Roseburia*, *Turicibacter*, and *Coprococcus* (Figure 5), suggesting the conservation of host genetic effect on the relative abundance of these bacteria across mammalian species.

### GWAS Identified Host Genomic Loci Affecting Microbial Composition of Porcine Cecum Lumen and Feces

**FIGURE 6:**
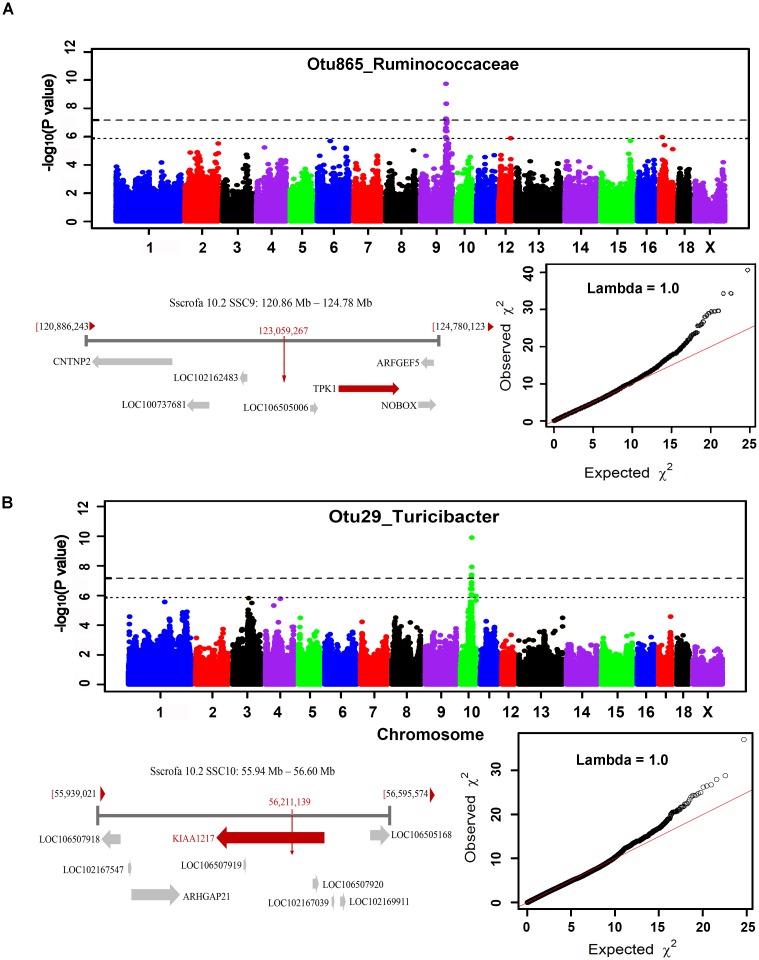
GWAS results and candidate gene annotation for *Ruminococcaceae* **(A)** and *Turicibacter* **(B).** For Manhattan plots, X-axis shows chromosomal positions. Y-axis shows –log10 *P*-values from GWAS. The horizontal dotted lines indicate the thresholds of genome-wide and suggestive significance level. Candidate gene closest to the top SNP for each locus was identified based on Porcine reference genome assembly 10.2 in Ensemble. The red vertical lines indicate the positions of the top SNPs. The red horizontal arrow bar represents the closest gene.

The numbers of experimental pigs, SNPs, OTUs and microbial taxa used for GWAS are shown in Table 1. In fecal samples, after quality control, a total of 1,411 non-redundant OTUs and 153 taxa were used for GWAS analysis. We identified a total of 40 significant associations, including 30 associations achieving genome-wide significance level (*P* < 6.65 × 10^-8^) and the other 10 associations at the suggestive significance level (*P* < 1.33 × 10^-6^). The significant associations for taxa and OTUs are shown in Table 2 and Supplementary Table S3, respectively. The most significant association was identified at SSC9: 123.06 Mb for Ruminococcaceae (OTU865; *P* = 1.84 × 10^-10^), where the most significant SNP was nearest to *TPK1* (Figure 6A). Twenty-three of these 40 associations were distributed across seven heritable bacterial taxa (*h*^2^ > 0.15), and there were also 17 associations with 10 taxa that appeared not to be significantly heritable.

**Table 1:** The numbers of experimental animals, SNPs, OTUs and bacterial taxa, and the significance threshold used for GWAS.

Population	Animal	SNP	Genome-wide significance	Suggestive significance	Taxon		OTU	
						
					Quantitative	Two-part	Quantitative	Two-part
Erhualian	227	731,411	6.84 × 10^-8^	1.37 × 10^-6^	76^a^	92^b^	105	1072
Bamaxiang	235	751,802	6.65 × 10^-8^	1.33 × 10^-6^	117^c^	36^d^	258	1143


*^*a*^Phylum 6, Class 11, order 10, family 19, Genus 26, and Species 4.*

*^*b*^Phylum 6, Class 11, order 10, family 16, Genus 52, and Species 13.*

*^*c*^Phylum 13, Class 15, order 28, family 28, Genus 27, and Species 6.*

*^*d*^Phylum 2, class 3, order 6, family 8, Genus 12, and Species 5.*

**Table 2:** GWAS results for bacterial taxa.

Taxonomy	Chromosome	Interval (Mb)	Number of significant SNPs^a^	*P*-value^b^	Nearest gene^c^	Gene function annotation
**CECUM LUMEN**						
Christensenellaceae	17	29.08–29.13	6	2.47E-08	PCSK2	Decreased circulating glucose level; decreased circulating insulin level
Peptococcaceae	5	54.91–59.76	8	3.57E-07	SLC15A5	Abnormal thyroid hormone level; immune system phenotype
**FECES**						
Acholeplasmatales	9	117.41–118.33	5	7.30E-08	PIK3CG	Involved in the immune response
Prevotella copri	15	100.34–112.02	19	1.28E-09	ZNF804A	Regulates the expression of genes involved in cell adhesion
Prevotella copri	13	134.63–134.66	13	1.75E-08	SST	Immune system phenotype; increased circulating insulin level


*^*a*^The number of SNPs achieving genome-wide significance level.*

*^*b*^P-values of the most significant SNP.*

*^*c*^The genes closest to the most significant SNPs (Sscrofa 10.2).*

In cecum luminal samples, 1,177 OTUs and 168 bacterial taxa were used for GWAS. We identified a total of 34 significant associations related to 17 taxa, including 18 associations achieving genome-wide significance level (*P* < 6.84 × 10^-8^) and 16 associations at the suggestive significance level (*P* < 1.37 × 10^-6^) (Table 2 and Supplementary Table S4). Of these 17 taxa, more than 70.0% (12 taxa) belonged to the heritable taxa having *h*^2^ > 0.15. The most significant association was identified at SSC10: 56.21 Mb for *Turicibacter* (OTU29) (*P* = 1.27 × 10^-10^), where the most significant SNP were located on *KIAA1217* (Figure 6B).

### Host Candidate Genes Associated With Gut Microbiota

**FIGURE 7:**
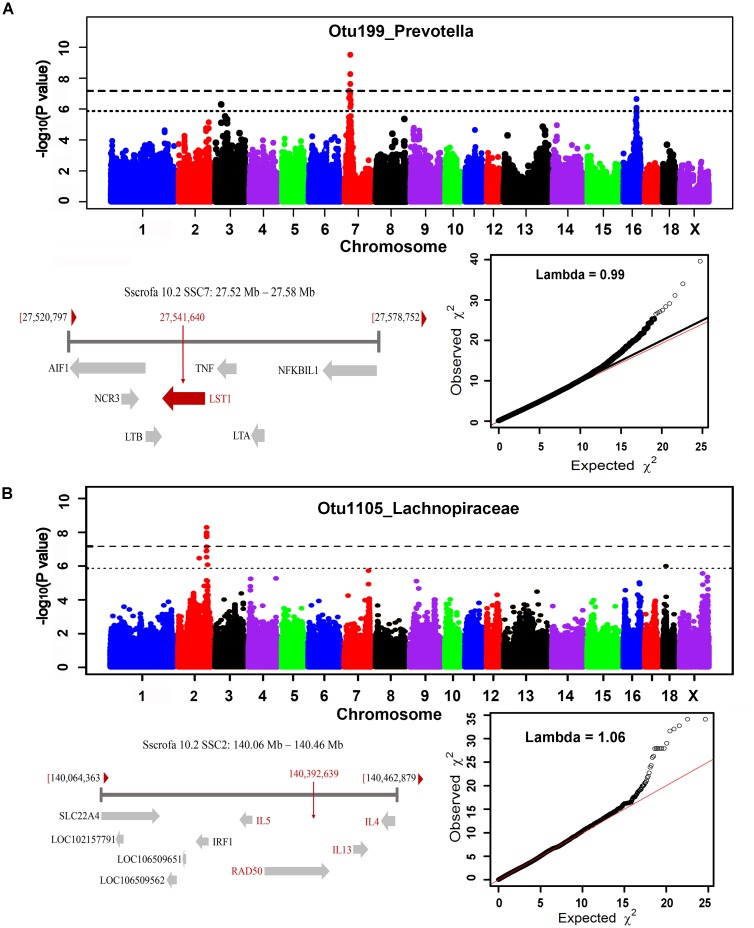
MHC region on SSC7 **(A)** and interleukin gene family region on SSC2 **(B)** that were identified to associate *Prevotella* and *Lachnospiraceae*. For Manhattan plots, X-axis shows chromosomal positions. Y-axis shows –log10 *P*-values from GWAS. The horizontal dotted lines indicate the thresholds of genome-wide and suggestive significance level. The red horizontal arrow bar represents the closest gene.

In order to identify host candidate genes influencing the gut microbial composition, we retrieved the genes nearest to the most significant SNPs for all genomic loci identified above based on porcine reference genome assembly (Sscrofa 10.2). Thirty-seven candidate genes and 1 LincRNA were identified for the associations in stool samples. Functional annotation of these candidate genes based on GeneCards and MGI database (Eppig et al., 2015) found that 13 out of these 37 candidate genes are related to metabolism, 13 genes are associated with immune functions, and 5 genes are related to signal transduction (Table 2 and Supplementary Table S3). Interestingly, the MHC region (SSC7: 27.54 Mb) was identified to associate the abundance of *Prevotella*. As we have well known, MHC region is related to host immune response. The top SNP was located on *LST1* (Figure 7A). *LST1* encodes a membrane protein that inhibits the proliferation of lymphocytes. The expression of *LST1* is enhanced by lipopolysaccharide, interferon-gamma and bacteria (Heidemann et al., 2014). Proinflammatory expression of *LST1* occurs in the setting of human IBD (Heidemann et al., 2014). Moreover, *TNF*, *LTB*, and *LTA* that are related to host immune response are also located within this region.

A total of 35 candidate genes were identified for the associations in cecum luminal samples (Table 2 and Supplementary Table S4). Functional classification found that 12 out of these 35 genes are related to metabolism (including obesity), and 12 candidate genes are associated with immune functions. There were also three and two candidate genes having been reported to associate signal transduction and cell adhesion, respectively. One of the interesting loci was identified at SSC2: 140.39 Mb, which was significantly associated with Lachnospiraceae (OTU1105, *P* = 5.18 × 10^-9^) and where the top SNPs was located within interleukin gene family region (*IL4*, *IL5*, and *IL13*) (Figure 7B).

## Discussion

The studies in humans and mice have indicated that host genetics influences gut microbial composition (McKnite et al., 2012; Org et al., 2015b; Davenport, 2016; Goodrich et al., 2016a). However, what extent to the host genetic effect on the gut microbial composition has still been controversial, and common variants in host genome contributing to the composition of gut microbiota are largely unknown. Especially, to the best of our knowledge, there is no study about the effect of host genetics on the microbial compositions of different gut compartments. In this study, we separately investigated the host genetic effect on the gut microbial compositions in porcine stool and cecum lumen samples through comparison of the β-diversity of gut microbiota among full-sibs, half-sibs and unrelated individuals, heritability estimate, and GWAS. To our knowledge, this is the first report in pigs about host genotypes shaping gut microbiome. For the first time, we evaluated the host genetic effect on the microbial composition of cecum, where the microbiota had the greatest diversity and complexity (Looft et al., 2014).

Although all experimental pigs were raised in the same farm house and provided with the same commercial formula diets (Materials and Methods), a significant difference of microbial community structure was observed between cecum lumen and feces. Previous report suggested that microbiota in stool is mostly from the colon and luminal microbes (Eckburg et al., 2005). The other factor should be the different genetic background between two experimental pig populations. Tremendous difference of microbial composition was also observed among individuals within a population raised in the uniform feeding conditions. Concordance with the observation in humans (Goodrich et al., 2014), a higher similarity of microbial community structures was observed in full-sib members than in unrelated pigs. We also found a significant correlation between the microbial composition-based kinship and the host SNP-based kinship. These results suggested the host genetic effect on the gut microbial composition in pigs.

Heritability estimates identified tens of heritable bacterial taxa in the two types of samples (Supplementary Table S2). Interestingly, some of these heritable taxa were evidenced in human (Davenport et al., 2015; Goodrich et al., 2016a) and mouse (O’Connor et al., 2014; Org et al., 2015b; Figure 5), suggesting that the host genetic effect on microbial composition is conserved and widespread in mammals. Previous reports in humans suggested that the majority of the heritable taxa belongs to Firmicutes (Goodrich et al., 2016b) and Proteobacteria (Davenport et al., 2015), whereas Bacteroidetes are generally not heritable. In this study, we found that 47.17% (25/53) and 50.0% (24/48) of the heritable taxa in cecum lumen and feces samples belong to Firmicutes, and 16.98% (9/53) and 12.5% (6/48) were from Proteobacteria. However, we also identified some heritable taxa from Bacteroidetes (9/53 in cecum lumen and 12/48 in feces).

The significant associations between host genotypes and relative abundances of the commonly identified taxa were not evidenced each other in the two types of samples. This should attribute to the reasons: (1) As described above, the samples were harvested from different gut compartments (cecum vs. stool); (2) Similar to other complex traits, population heterogeneity exists for host genetic effect on gut microbiota in different breeds. It was a limitation for this study that we could not harvest the samples from the same gut site of different pig populations or from the different gut sites of the same pig population. However, more than 26.5% of the heritable taxa were commonly identified in both cecum lumen and feces (Figure 4). Furthermore, the numbers of OTUs and taxa that were detected the significant associations, the numbers of genomic loci/candidate genes identified in the GWAS, and the functional categories of candidate genes were similar between cecum lumen and feces.

Non-redundant OTUs and taxa were used for GWAS. Although there were some OTUs annotated to the same bacterial family or genus, their relative abundances were associated with the different genomic loci. As we have known, OTUs were clustered based on the 97% sequence similarity of tags. These OTUs may belong to different bacterial species or strains. In many cases, different bacterial species or strains from the same genus should interact with different host genes. As an example reported previously, the strains K88, K99, 987P, and F41 of Enterotoxigenic *Escherichia coli* (ETEC) are pathogens of diarrhea in neonatal and weaned pigs (Nagy and Fekete, 2005). Different intestinal protein receptors are responsible for these ETEC strains, such as histone H1 proteins 7 for 987P (Zhu et al., 2005), MUC13 for K88 (Ren et al., 2012) and the undetermined receptors for K99 and F41. On the other hand, some host genomic loci were identified to affect the relative abundance of bacterial taxa, suggesting the common effect of a genomic locus on a functionally similar taxon (e.g., the same intestinal receptor for a bacterial genus).

Previous studies across mammalian species revealed a common theme that host genes involved in immune regulation and barrier defense are associated with microbiome variation (Horton et al., 2014; Davenport, 2016). Interestingly, of the 71 candidate genes identified in the two types of samples, nearly 35.2% (25/71) are related to immune functions and regulation. There were also 35.2% of candidate genes (25/71) related to metabolism, such as circulating glucose and insulin level, obesity, and so on (Table 2 and Supplementary Tables S3, S4). In humans, genes related to metabolism also emerge to involve in microbiome variation (Goodrich et al., 2016a). These results imply that the mechanisms about host genetic effect on gut microbiome are similar across mammalian species. *SHH* and *FGF20* influencing intestinal morphology in humans and knockout mice (Ramalho-Santos et al., 2000; Danopoulos et al., 2017) were associated with the bacterial abundance in this study (Supplementary Tables S3, S4). Abnormal intestine morphology should influence the interaction between microbiota and mucosal surfaces that underlies genetic associations. More interestingly, *EHBP1* having been reported to associate Crohn’s disease in Ashkenazi Jewish (Kenny et al., 2012) was identified to relate the abundance of Bacteroidales (Supplementary Table S4).

In summary, we systematically evaluated the effect of host genetics on porcine gut microbial composition separately in the samples harvested from cecum and stool. We identified tens of heritable bacterial taxa in heritability estimation. Functional classifications of candidate genes identified in the GWAS for microbial taxa are mainly associated with metabolism, immunity functions and signal transduction. The high similarity of heritable taxa and functional categories of candidate genes among pig, human and mouse suggests the conservation of the host genetic effect on gut microbiome across mammalian species. The results from this study gave another support that host genetics contributes to the variation of gut microbial composition and provided the basic data for further investigating the interaction between host genotypes and gut microbiome through isolating the causative mutations.

## Author Contributions

LH conceived and designed the experiments, revised the manuscript. CC conceived and designed the experiments, analyzed the data, wrote and revised the manuscript. XH performed the experiments, analyzed the data and wrote the part of manuscript. SF performed the experiments and took part in the data analysis. HY, MH, and YZ collected the samples and extracted the DNA. All authors read and approved the final manuscript.

## Conflict of Interest Statement

The authors declare that the research was conducted in the absence of any commercial or financial relationships that could be construed as a potential conflict of interest.

## Supplementary Material

Figure S1 Comparisons of phylogenetic diversity of microbial composition among full-sibs, half-sibs and unrelated individuals by weighted Unifrac analysis using the QIIME (v 1.9).

Table S1 Summary of 16S rRNA gene sequencing data.

Table S2 Heritability of microbial taxa estimated in cecum lumen and fecal samples.

Table S3 Host genomic loci and candidate genes identified by GWAS for OTUs in fecal samples.

Table S4 Host genomic loci and candidate genes identified by GWAS for OTUs in cecum luminal samples.

## References

[B1] Aulchenko Y. S. Ripke S. Isaacs A. van Duijn C. M. (2007). GenABEL: an R library for genome-wide association analysis. *Bioinformatics* 23 1294–1296. 10.1093/bioinformatics/btm108 17384015

[B2] Benjamini Y. Hochberg Y. (1995). Controlling the false discovery rate: a practical and powerful approach to multiple testing. *J. R. Stat. Soc. Ser. B* 57 289–300.

[B3] Benson A. K. Kelly S. A. Legge R. Ma F. Low S. J. Kim J. (2010). Individuality in gut microbiota composition is a complex polygenic trait shaped by multiple environmental and host genetic factors. *Proc. Natl. Acad. Sci. U.S.A.* 107 18933–18938. 10.1073/pnas.1007028107 20937875 PMC2973891

[B4] Blekhman R. Goodrich J. K. Huang K. Sun Q. Bukowski R. Bell J. T. (2015). Host genetic variation impacts microbiome composition across human body sites. *Genome Biol.* 16:191. 10.1186/s13059-015-0759-1 26374288 PMC4570153

[B5] Bonder M. J. Kurilshikov A. Tigchelaar E. F. Mujagic Z. Imhann F. Vila A. V. (2016). The effect of host genetics on the gut microbiome. *Nat. Genet.* 48 1407–1412. 10.1038/ng.3663 27694959

[B6] Camarinha-Silva A. Maushammer M. Wellmann R. Vital M. Preuss S. Bennewitz J. (2017). Host genome influence on gut microbial composition and microbial prediction of complex traits in pigs. *Genetics* 206 1637–1644. 10.1534/genetics.117.200782 28468904 PMC5500156

[B7] Campbell J. H. Foster C. M. Vishnivetskaya T. Campbell A. G. Yang Z. K. Wymore A. (2012). Host genetic and environmental effects on mouse intestinal microbiota. *ISME J.* 6 2033–2044. 10.1038/ismej.2012.54 22695862 PMC3475380

[B8] Cole J. R. Chai B. Farris R. J. Wang Q. Kulam S. A. McGarrell D. M. (2005). The Ribosomal Database Project (RDP-II): sequences and tools for high-throughput rRNA analysis. *Nucleic Acids Res.* 33 D294–D296. 10.1093/nar/gki038 15608200 PMC539992

[B9] Costello E. K. Lauber C. L. Hamady M. Fierer N. Gordon J. I. Knight R. (2009). Bacterial community variation in human body habitats across space and time. *Science* 326 1694–1697. 10.1126/science.1177486 19892944 PMC3602444

[B10] Danopoulos S. Schlieve C. R. Grikscheit T. C. Al Alam D. (2017). Fibroblast growth factors in the gastrointestinal tract: twists and turns. *Dev. Dyn.* 246 344–352. 10.1002/dvdy.24491 28198118

[B11] Davenport E. R. (2016). Elucidating the role of the host genome in shaping microbiome composition. *Gut Microbes* 7 178–184. 10.1080/19490976.2016.1155022 26939746 PMC4856462

[B12] Davenport E. R. Cusanovich D. A. Michelini K. Barreiro L. B. Ober C. Gilad Y. (2015). Genome-wide association studies of the human gut microbiota. *PLoS One* 10:e0140301. 10.1371/journal.pone.0140301 26528553 PMC4631601

[B13] De Filippo C. Cavalieri D. Di Paola M. Ramazzotti M. Poullet J. B. Massart S. (2010). Impact of diet in shaping gut microbiota revealed by a comparative study in children from Europe and rural Africa. *Proc. Natl. Acad. Sci. U.S.A.* 107 14691–14696. 10.1073/pnas.1005963107 20679230 PMC2930426

[B14] Desantis T. Z. Hugenholtz P. Larsen N. Rojas M. Brodie E. L. Keller K. (2006). Greengenes: chimera-checked 16S rRNA gene database and workbenchcompatible in ARB. *Appl. Environ. Microbiol.* 72 5069–5072. 10.1128/AEM.03006-05 16820507 PMC1489311

[B15] Devlin B. Roeder K. Wasserman L. (2001). Genomic control, a new approach to genetic-based association studies. *Theor. Popul. Biol.* 60 155–166. 10.1006/tpbi.2001.1542 11855950

[B16] Eckburg P. B. Bik E. M. Bernstein C. N. Purdom E. Dethlefsen L. Sargent M. (2005). Diversity of the human intestinal microbial flora. *Science* 308 1635–1638. 10.1126/science.1110591 15831718 PMC1395357

[B17] Eppig J. T. Blake J. A. Bult C. J. Kadin J. A. Richardson J. E. Mouse Genome Database Group (2015). The Mouse Genome Database (MGD): facilitating mouse as a model for human biology and disease. *Nucleic Acids Res.* 43 D726–D736. 10.1093/nar/gku967 25348401 PMC4384027

[B18] Goodrich J. K. Davenport E. R. Beaumont M. Jackson M. A. Knight R. Ober C. (2016a). Genetic determinants of the gut microbiome in UK twins. *Cell Host Microbe* 19 731–743. 10.1016/j.chom.2016.04.017 27173935 PMC4915943

[B19] Goodrich J. K. Davenport E. R. Waters J. L. Clark A. G. Ley R. E. (2016b). Cross-species comparisons of host genetic associations with the microbiome. *Science* 352 532–535. 10.1126/science.aad9379 27126034 PMC5116907

[B20] Goodrich J. K. Waters J. L. Poole A. C. Sutter J. L. Koren O. Blekhman R. (2014). Human genetics shape the gut microbiome. *Cell* 159 789–799. 10.1016/j.cell.2014.09.053 25417156 PMC4255478

[B21] Heidemann J. Kebschull M. Tepasse P. R. Bettenworth D. (2014). Regulated expression of leukocyte-specific transcript (LST) 1 in human intestinal inflammation. *Inflamm. Res.* 63 513–517. 10.1007/s00011-014-0732-6 24682411

[B22] Horton M. W. Bodenhausen N. Beilsmith K. Meng D. Muegge B. D. Subramanian S. (2014). Genome-wide association study of *Arabidopsis thaliana* leaf microbial community. *Nat. Commun.* 5:5320. 10.1038/ncomms6320 25382143 PMC4232226

[B23] Hsiao E. Y. McBride S. W. Hsien S. Sharon G. Hyde E. R. McCue T. (2013). Microbiota modulate behavioral and physiological abnormalities associated with neurodevelopmental disorders. *Cell* 155 1451–1463. 10.1016/j.cell.2013.11.024 24315484 PMC3897394

[B24] Kenny E. E. Pe’er I. Karban A. Ozelius L. Mitchell A. A. Ng S. M. (2012). A genome-wide scan of Ashkenazi Jewish Crohn’s disease suggests novel susceptibility loci. *PLoS Genet.* 8:e1002559. 10.1371/journal.pgen.1002559 22412388 PMC3297573

[B25] Looft T. Allen H. K. Cantarel B. L. Levine U. Y. Bayles D. O. Alt D. P. (2014). Bacteria, phages and pigs: the effects of in-feed antibiotics on the microbiome at different gut locations. *ISME J.* 8 1566–1576. 10.1038/ismej.2014.12 24522263 PMC4817603

[B26] Magoc T. Salzberg S. L. (2011). FLASH: fast length adjustment of short reads to improve genome assemblies. *Bioinformatics* 27 2957–2963. 10.1093/bioinformatics/btr507 21903629 PMC3198573

[B27] McKnite A. M. Perez-Munoz M. E. Lu L. Williams E. G. Brewer S. Andreux P. A. (2012). Murine gut microbiota is defined by host genetics and modulates variation of metabolic traits. *PLoS One* 7:e39191. 10.1371/journal.pone.0039191 22723961 PMC3377628

[B28] Mi H. Muruganujan A. Casagrande J. T. Thomas P. D. (2013). Large-scale gene function analysis with the PANTHER classification system. *Nat. Protoc.* 8 1551–1566. 10.1038/nprot.2013.092 23868073 PMC6519453

[B29] Nagy B. Fekete P. Z. (2005). Enterotoxigenic *Escherichia coli* in veterinary medicine. *Int. J. Med. Microbiol.* 295 443–454. 10.1016/j.ijmm.2005.07.003 16238018

[B30] Navas-Molina J. A. Peralta-Sánchez J. M. González A. McMurdie P. J. Vázquez-Baeza Y. Xu Z. (2013). Advancing our understanding of the human microbiome using QIIME. *Methods Enzymol.* 531 371–444. 10.1016/B978-0-12-407863-5.00019-8 24060131 PMC4517945

[B31] O’Connor A. Quizon P. M. Albright J. E. Lin F. T. Bennett B. J. (2014). Responsiveness of cardiometabolic-related microbiota to diet is influenced by host genetics. *Mamm. Genome* 25 583–599. 10.1007/s00335-014-9540-0 25159725 PMC4239785

[B32] Org E. Mehrabian M. Lusis A. J. (2015a). Unraveling the environmental and genetic interactions in atherosclerosis: central role of the gut microbiota. *Atherosclerosis* 241 387–399. 10.1016/j.atherosclerosis.2015.05.035 26071662 PMC4510029

[B33] Org E. Parks B. W. Joo J. W. Emert B. Schwartzman W. Kang E. Y. (2015b). Genetic and environmental control of host-gut microbiota interactions. *Genome Res.* 25 1558–1569. 10.1101/gr.194118.115 26260972 PMC4579341

[B34] Pearson T. A. Manolio T. A. (2008). How to interpret a genome-wide association study. *JAMA* 299 1335–1344. 10.1001/jama.299.11.1335 18349094

[B35] Purcell S. Neale B. Todd-Brown K. Thomas L. Ferreira M. A. Bender D. (2007). PLINK: a tool set for whole-genome association and population-based linkage analyses. *Am. J. Hum. Genet.* 81 559–575. 10.1086/519795 17701901 PMC1950838

[B36] Ramalho-Santos M. Melton D. A. McMahon A. P. (2000). Hedgehog signals regulate multiple aspects of gastrointestinal development. *Development* 127 2763–2772. 10821773 10.1242/dev.127.12.2763

[B37] Ren J. Yan X. Ai H. Zhang Z. Huang X. Ouyang J. (2012). Susceptibility towards enterotoxigenic *Escherichia coli* F4ac diarrhea is governed by the MUC13 gene in pigs. *PLoS One* 7:e44573. 10.1371/journal.pone.0044573 22984528 PMC3440394

[B38] Rooks M. G. Garrett W. S. (2016). Gut microbiota, metabolites and host immunity. *Nat. Rev. Immunol.* 16 341–352. 10.1038/nri.2016.42 27231050 PMC5541232

[B39] Turnbaugh P. J. Ley R. E. Mahowald M. A. Magrini V. Mardis E. R. Gordon J. I. (2006). An obesity-associated gut microbiome with increased capacity for energy harvest. *Nature* 444 1027–1031. 10.1038/nature705414 17183312

[B40] Turpin W. Espin-Garcia O. Xu W. Silverberg M. S. Kevans D. Smith M. I. (2016). Association of host genome with intestinal microbial composition in a large healthy cohort. *Nat. Genet.* 48 1413–1417. 10.1038/ng.3693 27694960

[B41] Wang J. Thingholm L. B. Skieceviciene J. Rausch P. Kummen M. Hov J. R. (2016). Genome-wide association analysis identifies variation in vitamin D receptor and other host factors influencing the gut microbiota. *Nat. Genet.* 48 1396–1406. 10.1038/ng.3695 27723756 PMC5626933

[B42] Wang Q. Garrity G. M. Tiedje J. M. Cole J. R. (2007). Naive Bayesian classifier for rapid assignment of rRNA sequences into the new bacterial taxonomy. *Appl. Environ. Microbiol.* 73 5261–5267. 10.1128/AEM.00062-07 17586664 PMC1950982

[B43] Yang H. Huang X. Fang S. Xin W. Huang L. Chen C. (2016). Uncovering the composition of microbial community structure and metagenomics among three gut locations in pigs with distinct fatness. *Sci. Rep.* 6:27427. 10.1038/srep27427 27255518 PMC4891666

[B44] Zhou X. Carbonetto P. Stephens M. (2013). Polygenic modeling with Bayesian sparse linear mixed models. *PLoS Genet.* 9:e1003264. 10.1371/journal.pgen.1003264 23408905 PMC3567190

[B45] Zhou X. Stephens M. (2012). Genome-wide efficient mixed-model analysis for association studies. *Nat. Genet.* 44 821–824. 10.1038/ng.2310 22706312 PMC3386377

[B46] Zhu G. Chen H. Choi B. K. Del Piero F. Schifferli D. M. (2005). Histone H1 proteins act as receptors for the 987P fimbriae of enterotoxigenic *Escherichia coli*. *J. Biol. Chem.* 280 23057–23065. 10.1074/jbc.M503676200 15840569

