# Supplementary figures and images for: Contribution of Host Genetics to the Variation of Microbial Composition of Cecum Lumen and Feces in Pigs
Source: Front Microbiol. 2018 Oct 31;9:2626. doi: 10.3389/fmicb.2018.02626 (PMC6220110; doi:10.3389/fmicb.2018.02626)

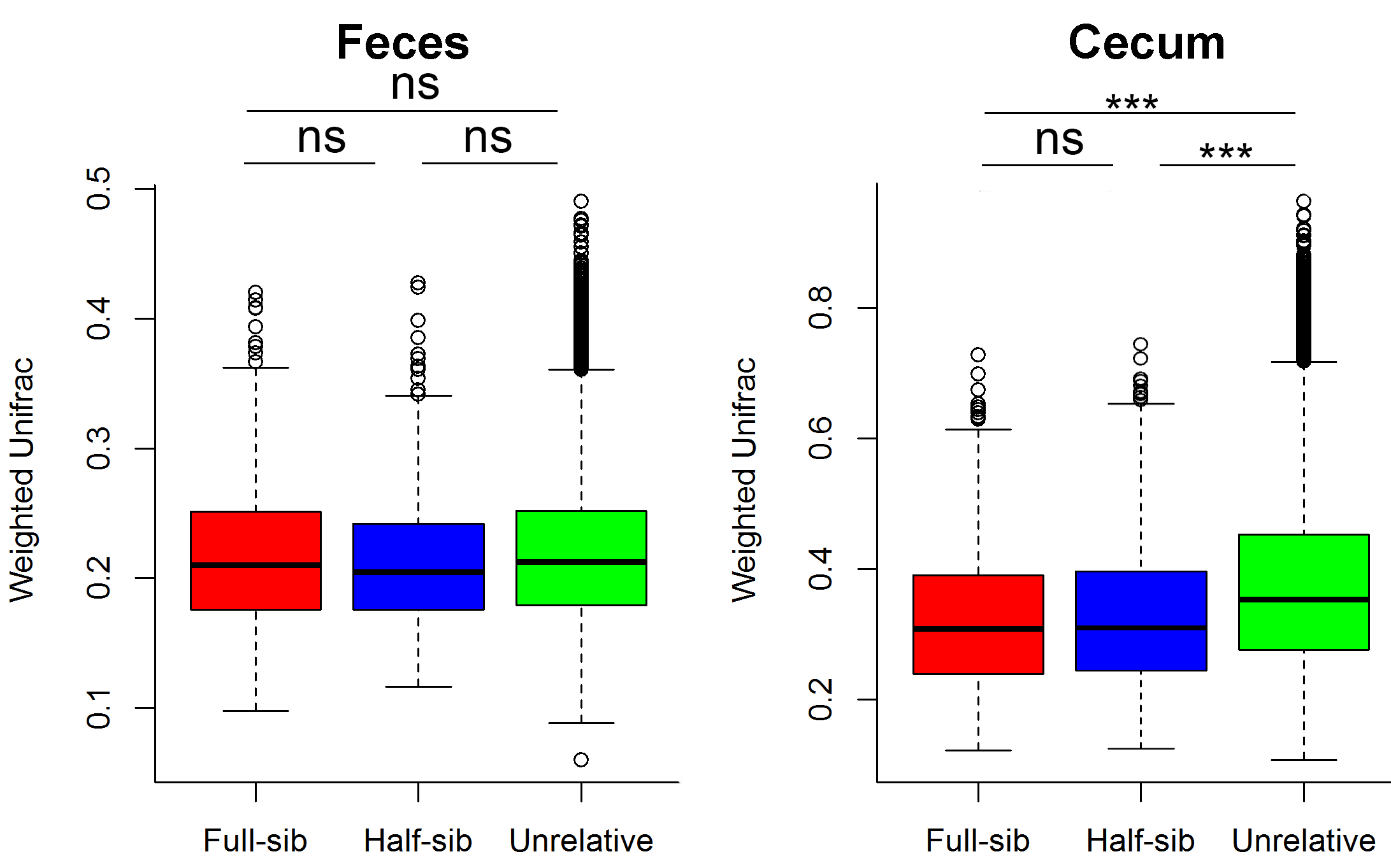

Supplement: Figure S1 — Comparisons of phylogenetic diversity of microbial composition among full-sibs, half-sibs and unrelated individuals by weighted Unifrac analysis using the QIIME (v 1.9). [file Image_1.TIF]
