# Supplementary material for: Contribution of Host Genetics to the Variation of Microbial Composition of Cecum Lumen and Feces in Pigs
Source: Front Microbiol. 2018 Oct 31;9:2626. doi: 10.3389/fmicb.2018.02626 (PMC6220110; doi:10.3389/fmicb.2018.02626)
Supplement: Table S1 — Summary of 16S rRNA gene sequencing data. [file Table_1.DOC]

**Table S1.** **Summary of 16S rRNA gene sequencing data**

| **Item** | **Cecum lumen** | | | **Feces** | | |
| --- | --- | --- | --- | --- | --- | --- |
|  | **Mean** | **Min** | **Max** | **Mean** | **Min** | **Max** |
| Clean read | 36,324 | 19,356 | 62,376 | 61,669 | 26,432 | 102,350 |
| Tag number | 18,162 | 9,678 | 31,188 | 22,174 | 12,947 | 38,673 |
| OTU number | 718 | 366 | 952 | 888 | 618 | 1,066 |
| Phylum number | 13 | 8 | 16 | 15 | 12 | 18 |
| Genus number | 90 | 56 | 116 | 80 | 73 | 83 |
